# *iMOSFLM*: a new graphical interface for diffraction-image processing with *MOSFLM*

**DOI:** 10.1107/S0907444910048675

**Published:** 2011-03-18

**Authors:** T. Geoff G. Battye, Luke Kontogiannis, Owen Johnson, Harold R. Powell, Andrew G. W. Leslie

**Affiliations:** a MRC Laboratory of Molecular Biology, Hills Road, Cambridge CB2 0QH, England

**Keywords:** data integration, graphical user interface, data processing, software

## Abstract

*iMOSFLM* is a graphical user interface to the diffraction data-integration program *MOSFLM*. It is designed to simplify data processing by dividing the process into a series of steps, which are normally carried out sequentially. Each step has its own display pane, allowing control over parameters that influence that step and providing graphical feedback to the user. Suitable values for integration parameters are set automatically, but additional menus provide a detailed level of control for experienced users. The image display and the interfaces to the different tasks (indexing, strategy calculation, cell refinement, integration and history) are described. The most important parameters for each step and the best way of assessing success or failure are discussed.

## Abstract 2

A new graphical user interface to the *MOSFLM* program has been developed to simplify the processing of macromolecular diffraction data. The interface, *iMOSFLM*, allows data processing *via* a series of clearly defined tasks and provides visual feedback on the progress of each stage.

## 1. Introduction

*MOSFLM* (Leslie, 2006 ▶) is a program to process diffraction data collected using the oscillation method (Arndt & Wonacott, 1977 ▶). A graphical user interface (GUI) for the program was developed in the 1990s based on a set of X11 routines provided by J. W. Campbell (Campbell, 1995 ▶). While this interface offered a high degree of functionality, the overall graphical quality was limited and the restrictions of the routines available made efficient and intuitive design difficult. A new Tcl/Tk-based GUI, *iMOSFLM*, has recently been developed to address these issues.

There are a variety of reasons why a graphical interface is valuable when processing diffraction data, and GUIs are a feature of many processing packages including *HKL*-2000 ▶ (Otwinowski & Minor, 1997 ▶), *SAINT* (Bruker Analytical X-­ray Systems, Madison, USA), d*TREK (Pflugrath, 1999 ▶) and *XGEN* (Howard, 2000 ▶). The ability to inspect the diffraction images, especially with the predicted diffraction pattern overlaid, is invaluable in identifying potential problems that might occur during subsequent processing. These arise for a wide variety of reasons, including poor spot shape, very high crystal mosaicity, multiple lattices, anisotropic diffraction, the presence of diffraction spots or rings owing to ice formation, shadows from backstops or experimental equipment, errors in the direct-beam position and zingers (bright pixels resulting from cosmic rays or radioactive decay events in the fibre-optic taper). Being able to continuously monitor the refined detector and crystal parameters as processing proceeds provides a means of assessing the stability of the refinement and identifying if and when problems occur.

The new interface was designed to provide an intuitive route to data processing, so that inexperienced users are guided in a logical fashion through the stages of data processing and alerted to potential problems. At the same time it was felt important to provide the full functionality of the *MOSFLM* program (available *via* a very large number of keywords in the command-line version) for more experienced users, but in an unobtrusive fashion. The overall structure of the GUI and a more detailed description of the individual tasks and their graphical output are given below.

## 2. Overall structure of the *iMOSFLM* GUI

*iMOSFLM* acts as a ‘front end’ to the *MOSFLM* program itself. *iMOSFLM* generates the *MOSFLM* commands for particular tasks, based on user input, and then passes these commands to *MOSFLM*, which carries out all the computation. The results of these tasks are then passed back to *iMOSFLM* for display, either on completion of the task (*e.g.* spot-finding, auto-indexing, strategy calculations) or while the task is still in progress (*e.g.* cell refinement and integration). *iMOSFLM* and *MOSFLM* are run as separate processes, with the *MOSFLM* process being started by *iMOSFLM*. This means that *iMOSFLM* always retains all the parameters relevant to a particular task, so that if *MOSFLM* encounters an error that causes it to fail, *iMOSFLM* can restart *MOSFLM* and the user can attempt to recover from the failure.

### 2.1. The *iMOSFLM* panes

**Figure 1:**
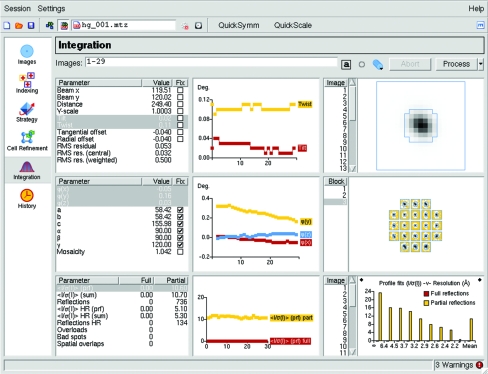
An overview of the *iMOSFLM* GUI. The Integration pane is shown and the icons for the various tasks are displayed in the vertical icon bar on the left-hand side of the window. Refined detector and crystal parameters are displayed graphically in the central upper and middle windows, respectively. Intensity statistics are displayed in the lower central and lower right-hand windows. The average spot profile for spots in the central region of the detector is shown in the upper right panel and the standard profiles for different regions of the detector are shown in the central right panel.

Each *iMOSFLM* task has its own pane where relevant parameters can be set and the results are displayed. The available tasks (Images, Indexing, Strategy, Cell Refinement, Integration and History) are listed on the vertical icon bar on the left-hand side of the GUI (Fig. 1 ▶) and can be selected by the user, but a particular icon will only become active (*i.e.* user-selectable) once any necessary preceding actions have been carried out. For example, indexing can only be selected once images have been added to the session using the Add Images button in the Images pane. This displays a file browser and selecting any image file will add all images that have the same filename template. Images with different filename templates can also be added but will be assigned to different sectors. The start and end oscillation angles of each image are listed and once an image has been processed the missetting angles (changes in crystal orientation relative to the initial orientation) are added. The cell, space group, mosaicity and mosaic block size are also listed in the Images pane (and are user-editable).

### 2.2. The Image Display window

**Figure 2:**
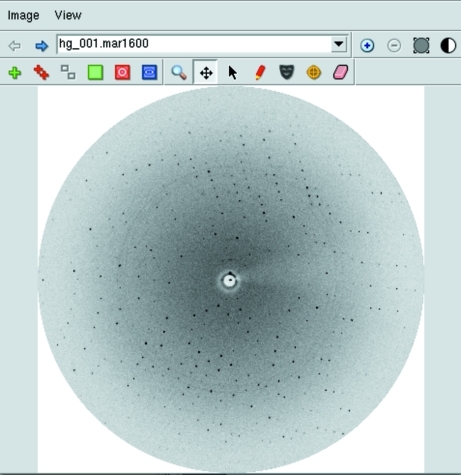
The *iMOSFLM* Image Display window. The functions of the buttons in the tool bar are explained in the main text.

As soon as one or more images have been added to the session, the first image will be displayed in the Image Display window (Fig. 2 ▶). Buttons in the tool bar control display of the direct-beam position, spots found (for indexing), predicted reflections, masked areas (*e.g.* the backstop shadow), spot search region and resolution limits, while other icons control zooming and panning, a selection tool, manual addition of spots, the definition of masked regions, the definition of the centre of a circle and the erasure of either masks or manually added spots. Additional icons control image zoom (about the current centre), image size and image contrast. Any image can be selected for display from a drop-down list. A useful feature of the zoom window is that the zoomed region is always pre­served when changing from one image to another. The View option in the menu bar allows the size of the image display to be changed, while the Tools option allows a reflection with particular indices to be located. The selection tool displays the resolution at any point in the image (when the Ctrl key is depressed) and when over a predicted reflection also displays the Miller indices. It can be used to make ‘drag-and-drop’ adjustments to the spot-search areas, resolution limits, direct-beam position and masked areas.

### 2.3. Drop-down menus

The Session and View menus are always accessible *via* the menu bar. The Session drop-down menu allows the user to start a new session, save the current session or load a previously saved session. A new session is started each time *iMOSFLM* is run or when selected by the drop-down menu. Saving a session stores all the information about the current state of the interface, including the images that have been read, the current values of all refineable parameters and processing options and the graphical information for all steps carried out during the session. The View menu allows access to the various settings dialogues that allow experimental and detector parameters to be defined (under Experiment Settings) and a large number of parameters and options influencing the processing stages to be set (under Processing Options). Some of these will be described in later sections.

## 3. The Indexing pane

**Figure 3:**
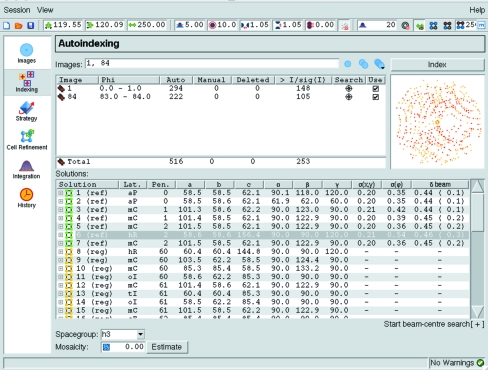
The *iMOSFLM* Indexing pane. Parameters that influence spot finding and indexing are shown in the tool bar. Details of the images used for indexing and the number of spots found and used are presented as a table and graphically. The list of possible indexing solutions is shown, with the preferred solution highlighted.

On selecting the Indexing task, *iMOSFLM* issues commands to find spots on two images: the first image in the series and the image that is as close as possible to a 90° rotation away from the first. Found spots are displayed both in the Image Display window and in a representation of the images in the Indexing pane (Fig. 3 ▶). Spots above the current threshold for use in indexing are shown as red crosses; those below the threshold are shown in yellow. Typically, indexing works best with a few hundred spots (in total). Additional images can be included in the indexing by entering the image numbers into the Images field or by selecting them from a drop-down list of all images. The images to be used in indexing, the number of spots found and the number of spots above the threshold are displayed. Individual images can be deselected by clicking the Use box, but can subsequently be included without repeating the spot search by selection from the drop-down list of all images. Auto-indexing, which is carried out by clicking the Index button, uses an FFT-based auto-indexing algorithm to determine the crystal lattice (Steller *et al.*, 1997 ▶; Powell, 1999 ▶). If successful, auto-indexing produces a list of possible solutions sorted on penalty. Typically, there will be a group of solutions with low penalty followed by a series of solutions with much higher penalty values (unless the crystal is triclinic, in which case there may only be a single solution with low penalty). *MOSFLM* selects a solution based on a simple analysis of the penalty and the metric symmetry. The preferred solution is highlighted and the prediction is displayed on the image display. At this stage, only information about the lattice shape is available (based on observed spot positions) and therefore the assignment of symmetry (above triclinic) is an assumption that needs to be tested after images have been integrated.

If the appropriate option is set in the Processing Options dialogue, the indexing and mosaicity-estimation processes will be carried out automatically after spot finding without the requirement to click the Index button.

### 3.1. Spot-finding parameters

Parameters that control spot finding are listed in the Spot finding tab of the Processing Options dialogue. The search area is set by default to be between circles of radii corresponding to 5 and 95% of the radius of the inscribed circle centred on the direct-beam position, but this can be adjusted graphically with the spot-finding button on the Image display or by setting the values explicitly. The default threshold value for a pixel to be considered part of a spot is set to 5.0σ above the background (where σ is determined by counting statistics) and a variety of rejection criteria are applied to distinguish true Bragg spots from noise features in the image. These include a minimum number of pixels, minimum and maximum sizes (mm), a minimum r.m.s. variation of pixel values within the spot, a maximum anisotropy in spot dimensions, a minimum spot separation and a maximum peak separation within spots. The default values have been optimized for images collected on synchrotron beamlines, where the spots tend to be smaller than for images collected on a laboratory source. For the latter, if the spots are large and diffuse then better results can be obtained by decreasing the threshold (*e.g.* to 2.0σ), increasing the minimum number of pixels to 20–30, reducing the r.m.s. spot variation to 1.0 and setting the minimum spot separation to values around 1.5 mm (although this is best set to the actual size of the diffraction spots in the image).

Two algorithms are available for determining the local X-­ray background. The simpler one assumes that the background is circularly symmetric about the direct-beam position and determines the background in a radial stripe 50 pixels wide. The orientation of this stripe is at 90° to the direction of the rotation axis in order to avoid any shadow arising from a solid backstop support (on the assumption that these are normally aligned parallel to the rotation axis), but this can be changed by ‘drag and drop’ in the Image display or *via* the Spot finding tab. For tiled detectors, this stripe is automatically offset to avoid using pixels in the gaps between tiles for the initial estimate of the radial background. The second and generally preferred algorithm uses a local background calculation, which by default finds the background in boxes of size 50 × 50 pixels. In some cases, the local background method results in spurious spots being located close to sharp shadows in the image (*e.g.* owing to the backstop support). These are normally below the threshold for use in auto-indexing and can be ignored, but reducing the size of the local background region (*e.g.* to 20 × 20 pixels) will often eliminate these spurious spots. Even when the local background method is being used, an initial radial stripe background is determined to set parameters associated with the background determination, so it is important that this stripe (which can be displayed on the image with the spot-finding search area button) does not lie over a large shadow on the detector.

The inclusion of spots arising from crystalline ice can easily result in the failure of auto-indexing. To avoid this problem, spots within narrow resolution shells centred on the principal reflections of hexagonal crystalline ice are automatically excluded. In addition, if the diffraction is very weak the resolution limit for spot finding is automatically reduced to 4.5 Å in order to avoid including any noise features that might occur at higher resolution (*e.g.* owing to zingers) and the minimum spot size is also decreased. If there are only a few true Bragg spots present, the inclusion of only a small number of noise spots can lead to failure of the indexing. Finally, the intensity threshold for spots to be included in the auto-indexing is automatically set to 5, 10 or 20 depending on the strength of the diffraction (on the last image processed), which also decreases the chance of indexing failure. All of these options can be overridden in the Processing Options dialogue.

### 3.2. Indexing parameters

Only two user-definable parameters influence the indexing algorithm. These are the threshold for spots to be included in indexing (as mentioned in §[Sec sec3.1]3.1) and the maximum cell edge. The default value for the latter is set to the real-space distance corresponding to the closest two spots to be used in indexing, but this can be too high if spurious spots are above the indexing threshold or if a second lattice is present.

### 3.3. Judging the success of the indexing

The most reliable way of assessing whether the chosen solution is correct is by inspecting the resulting prediction, preferably on all the images used in indexing and, if available, also on some images not used in indexing. The predicted reflections should agree with the observed Bragg spots both in position and in terms of the general appearance of the lunes (the typically crescent-shaped or sometimes approximately circular regions of spots on the image, each of which contains reflections from a different plane in reciprocal space). Note that unless the mosaicity has been estimated (see §[Sec sec3.7]3.7) not all observed spots are predicted. Different solutions in the indexing results table can be selected to check the corresponding predictions. A correct solution will normally have a penalty lower than 20, provided that the values for the experimental parameters (direct-beam position, distance and wavelength) are correct. Another useful indicator is the positional residual, denoted σ(x,y) in the table, which is the r.m.s. difference between the observed and calculated spot positions. Typical values are 0.1–0.2 mm for synchrotron images and 0.2–0.3 mm for images collected using a laboratory source (where the spots are larger). However, if the spots are split or very irregular in shape this value can be as high as 1–1.5 mm for the correct solution and therefore it is not possible to define a cutoff value that is applicable in all cases. Errors in the experimental parameters may also produce larger values. The positional residual can be a useful indicator of the presence of pseudosymmetry. If, for example, the symmetry is monoclinic but with a β angle close to 90°, the automatically chosen solution will probably be the (pseudo) ortho­rhombic one. If close inspection reveals that there is a monoclinic solution with a positional residual that is more than 0.1 mm less than that for the orthorhombic solution, it is very probable that the monoclinic solution is correct. Choosing the correct solution is important when selecting a data-collection strategy, but the only way to be confident of the correct Laue group is to collect and integrate images corresponding to a small rotation (*e.g.* 3–10°) and then to run *POINTLESS* (Evans, 2011 ▶; see §[Sec sec6.7]6.7).

### 3.4. Common causes of indexing failure

#### 3.4.1. Errors in direct-beam coordinates

The most common cause of indexing failure is having incorrect values for the direct-beam coordinates (these are read from the image-file header). The direct-beam position is indicated in the Image display (as a green cross) and so it is immediately obvious if this is seriously in error (*e.g.* not within the backstop shadow). The beam position can be adjusted manually (using the selection tool to drag and drop) and in favourable cases (small backstop shadow, clear lune definition) it can easily be positioned close enough to the correct position to allow indexing. If ice rings are present a tool is available in the Image display to determine the centre of the ring (and hence the direct-beam position) by circle-fitting a small number of points on a ring (this assumes that the face of the detector is normal to the X-ray beam, *i.e.* there is no 2θ offset). The accuracy required for the beam coordinates depends on the cell dimensions, with larger unit cells requiring more accurate coordinates. To avoid mis-indexing, the coordinates need to be known to an accuracy corresponding to less than one half of the spot separation for the longest cell axis. For cell axes longer than about 250 Å it can be very difficult to detect from the results of the indexing if the pattern is mis-indexed by one index along the long axis. However, integration of even a small rotation range and subsequent symmetry detection with *POINTLESS* will immediately detect this error, as even Friedel pairs will give poor agreement. As an alternative to manually defining the direct-beam position a two-dimensional grid search can be carried out where, by default, the beam coordinates will be varied by ±1.0 mm in 0.5 mm steps (these parameters can be changed in the Processing Options dialogue) and the indexing carried out for each new position. The positional residual and consistency between the refined beam coordinates can be used to select the most probable correct beam position. This procedure works best if at least two images (preferably widely separated in rotation angle) are used.

#### 3.4.2. Problem cases

It is not uncommon for images to show evidence of multiple lattices or for the second image used for indexing to show the results of severe radiation damage. In such cases, selecting a single (less problematic) image can result in successful indexing, while inclusion of both images fails. In the case of multiple lattices, it may only be necessary to increase the intensity threshold for spots to be included in the indexing, with values of up to 100 sometimes being necessary. The same approach works well for crystals with very high mosaicity (no distinct lunes visible). In marginal cases, usually those with very weak diffraction resulting in very few spots above the threshold, varying the threshold slightly from the default value can make the difference between failure and success. The use of more than two images can also be helpful in difficult cases, ideally with the additional images well separated in rotation angle Φ from those already being used. All of these modifications to the default behaviour are readily achieved *via* the Indexing pane.

Another possible cause of indexing failure, especially if the diffraction is strong and there is no evidence of other problems, is that the direction of spindle rotation is opposite to that conventionally used. This is the case on a number of synchrotron beamlines. In this case, indexing should be successful when only a single image is used, but if the next image in the series is inspected the prediction will not match the observed spots. This can be dealt with checking a box in the Detectors tab of the Experiment Settings dialogue and repeating the spot search for all images used in indexing to ensure that the correct Φ values are assigned.

### 3.5. Other indexing issues

Caution is required if only a single image is used for indexing since for low-symmetry space groups it is possible to find an indexing solution that will correctly predict one image but will not correctly predict images that differ significantly in Φ. This is why *iMOSFLM* always uses two images by default.

When inspecting the predicted pattern, it is also important to check if pseudo-centring or lattice repeats have resulted in a particular class of reflections being systematically weak. If only strong reflections are used in the indexing, the resulting unit cell will be too small to predict the weak reflections and the indexing should be repeated with a reduced intensity threshold.

### 3.6. Choice of space group

If the correct space group is known, it can be selected from a drop-down menu. If it is not known, the lowest symmetry should be assumed until the data have been integrated and the symmetry assessed with *POINTLESS* (see §[Sec sec6.7]6.7). The selection of the space group will only affect the strategy calculation and will have no effect whatever on cell refinement or data integration.

### 3.7. Mosaicity estimation

**Figure 4:**
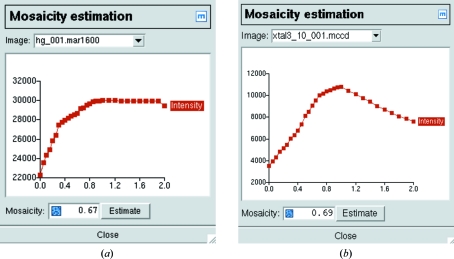
Mosaicity estimation. The total intensity for all predicted spots is plotted as a function of the mosaic spread. (*a*) In most cases the total intensity will reach a plateau at the correct value for the mosaic spread. (*b*) With large unit cells (or large oscillation angles) the total intensity can drop rather than plateau because spatially overlapping spots are not integrated.

An initial estimate of the crystal mosaicity is obtained (Rossmann, 1979 ▶) by integrating the (first) image with different values for the mosaic spread and selecting the value for which the total intensity of all predicted spots reaches a plateau value (Fig. 4 ▶ *a*). If the unit cell is large, the total intensity will drop for large values of the mosaic spread as some reflections become flagged as overlapped and are not integrated (Fig. 4 ▶ *b*). The mosaicity is refined during cell refinement and data integration, so the accuracy of this initial estimate is not critical.

#### 3.7.1. The mosaic block size

**Figure 5:**
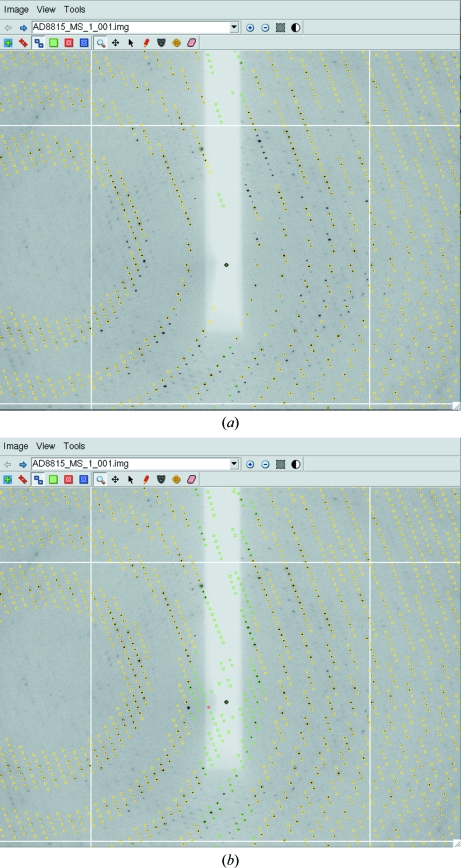
The effect of the mosaic block size on the predicted diffraction pattern. Reducing the mosaic block size effectively increases the apparent mosaic spread at low resolution with little or no effect at high resolution. (*a*) 100 µm mosaic block size. (*b*) 2 µm mosaic block size.

If the mosaic block size of the crystal is very small (less than a few micrometres) this has the effect of changing the apparent crystal mosaicity as a function of resolution (Nave, 1998 ▶; Juers *et al.*, 2007 ▶). In practice, this can give rise to an apparent mosaic spread of one or two degrees at low resolution compared with a much smaller value at high resolution. This can be modelled by reducing the mosaic block size (default value 100 µm) so that the predictions match the observed pattern at both low and high resolution (Fig. 5 ▶). This is performed manually by entering different values for the mosaic block size in the Image pane, but is best performed after cell refinement (see §[Sec sec5]5) when the cell, mosaic spread and crystal missetting angles have been optimized.

## 4. The Strategy pane

**Figure 6:**
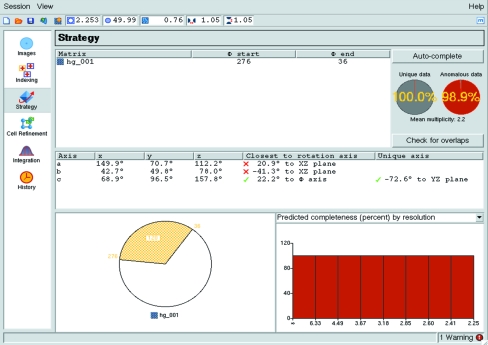
The *iMOSFLM* Strategy pane. Details are given in the main text.

Once an indexing solution has been found it is possible to access the Strategy pane (Fig. 6 ▶), which allows the calculation of an optimal geometrical data-collection strategy based on the Laue group and the crystal orientation defined in the indexing stage. On selecting the strategy task, values will be displayed for the completeness of the overall data and the anomalous data. These figures assume that the entire Φ range between the first and last images has been collected, which is not the case if only two initial screening images (normally separated by a 90° Φ rotation) have been taken in order to characterize the crystal. In these cases, the Auto-complete button should be used to calculate the best data-collection strategy. The default mode (Auto) will work out the Φ_start_ and Φ_end_ that will give a complete data set. Options are available to include data already collected from the same crystal in the same orientation or to optimize the completeness of anomalous data. In many space groups it is possible to collect data with high completeness (>95%) using a total Φ rotation that is significantly less than that strictly required for that Laue group, especially if the crystal is oriented so that none of the principal axes are aligned with the rotation axis. For example, in an orthorhombic space group a 60° rotation in two 30° segments is generally sufficient for 95% overall completeness (although the completeness will be less than this at low resolution). The option is therefore provided to collect the data in up to three distinct segments with a total rotation of between 30 and 90°. Various statistics on completeness and multiplicity as a function of resolution and total rotation angle are presented in graphical form. An interactive graphical representation of the suggested rotation segment(s) is displayed in the lower left area of the pane (Fig. 6 ▶) and by clicking it is possible to adjust the Φ_start_ and Φ_end_ values and recalculate the statistics.

Another button (Check for overlaps) will calculate the maximum oscillation angle per image that will avoid spatial overlaps, based on the current values for the resolution, minimum spot separation and crystal mosaicity, as a function of Φ for the suggested rotation range. Alternatively, the percentage of overlapped reflections can be calculated for different values of the oscillation angle. In both cases, the results are displayed graphically as histograms in the lower right area of the pane.

## 5. The Cell Refinement pane

**Figure 7:**
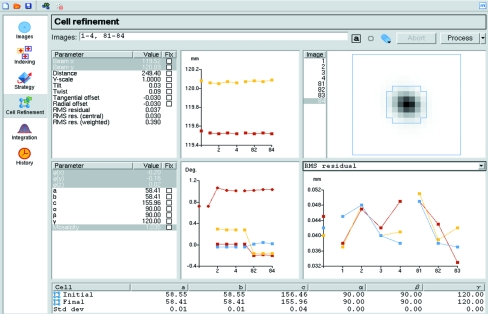
The *iMOSFLM* Cell Refinement pane. In this example the refined direct-beam coordinates are plotted in the upper central graph, the refined crystal missetting angles and mosaic spread are plotted in the lower central graph and the r.m.s. residual is plotted in the lower right graph, all as a function of image number. The average spot profile for spots in the central region of the detector is displayed in the upper right panel. The initial and refined cell parameters and an estimate of their standard uncertainties are given in the table.

The cell parameters determined from the auto-indexing step are based on spot positions and are limited in accuracy as they are highly correlated with the crystal-to-detector distance, which is not refined by default as, except for very high-resolution data, this distance is not well determined. The Cell Refinement task (Fig. 7 ▶) allows the refinement of cell parameters, crystal orientation and mosaicity based on a post-refinement procedure (Rossmann *et al.*, 1979 ▶; Winkler *et al.*, 1979 ▶; Leslie, 2006 ▶) that provides more accurate values provided that the resolution of the data is better than ∼3.5 Å. The procedure involves integrating a small number of segments of data (two by default, but three or more may give better results for triclinic or monoclinic symmetries). The optimal number of images in each segment, which depends on the oscillation angle and the mosaic spread, is calculated automatically. Two segments separated by 90° in Φ (or as close to 90° as possible) are chosen provided that the images are available. The distribution of the total intensity of partially recorded reflections across the images on which they lie, together with a model for the rocking curve, are used for the refinement. During integration of the images, the refined detector and crystal parameters are displayed graphically, together with the average spot profile for spots in the centre of the detector (Fig. 7 ▶). Any of these graphs can be expanded to fill the pane by using the mouse scroll wheel or a combination of shift and left mouse button. These graphs are useful in detecting any problems with the integration and will be described in more detail in §[Sec sec6.1]6.1. The detector parameters and crystal orientation are refined for every image, but the cell parameters are only refined following integration of all of the images. If there is a large shift in the cell parameters, all images are re-integrated and cell refinement is repeated, and this whole process is repeated to convergence (or a maximum of five times). On completion, the initial and final cell parameters and their estimated un­certainties are reported. The estimated uncertainties should normally be less than 0.1 Å in cell edges and 0.1° for cell angles. Graphs are produced of r.m.s. error in spot positions (referred to as r.m.s.d. below), the refined crystal-to-detector distance and the refined YSCALE parameter (a relative scale factor in the *Y* direction of the detector), both for each image and separately for each cycle of refinement. Indicators of a successful refinement are a decrease in the r.m.s.d. values, YSCALE values close to unity for all images (except for R-­AXIS IV and HTC image-plate detectors, for which the correct value is 0.995) and consistent detector distances for all images.

In situations where the data are too weak or the resolution is too low for successful post-refinement, the cell parameters obtained from the auto-indexing should be used, and in these cases including three or four images (widely separated in Φ) may improve the accuracy of the cell parameters.

## 6. The Integration pane

Although it is possible to integrate the data directly after auto-indexing, it is generally recommended that the cell parameters are refined first (as described in §[Sec sec5]5). This can provide a significant improvement in data quality, especially if the crystal-to-detector distance (read from the header of the image file) is significantly in error. A possible exception is in cases where the true Laue group is uncertain, for example a monoclinic space group with a β angle close to 90°. In such cases integration of 5–10° of data with the cell derived from auto-indexing, followed by symmetry assessment with *POINTLESS* (Evans, 2011 ▶), will allow determination of the true symmetry, which in turn will determine which cell parameters are to be refined.

The Integration pane (Fig. 1 ▶) allows control of data integration and in addition symmetry detection (*POINTLESS*) and preliminary scaling (*SCALA*; Evans, 2006 ▶). By default, the image display is not updated during integration as this adds a significant time overhead, but the option to turn image updating on or off during integration is accessible *via* a button on the tool bar. The results of the integration are written to a multi-record CCP4 MTZ file, which is assigned a filename based on the image filenames, but this can be reset if required. Other icons in the toolbar allow the rejection of all reflections lying in narrow resolution shells corresponding to crystalline ice and a ‘wait’ function that can be used to integrate images that have not yet been collected when processing is started. This latter option is useful when processing data that are being collected on a synchrotron beamline, as it allows processing of the entire data set to start before data collection is complete.

Data integration proceeds in blocks of images, with typically 5–10 images in a block. The detector parameters are refined for each image and the pixel values for the measurement boxes for all predicted spots are written to a scratch file. The standard profiles are formed using all the images in the block and each image is then integrated in turn. Normally, the results of integration are written to a single MTZ file that cannot be used (*e.g.* to run scaling on intermediate results) until pro­cessing is complete. The option is available in the Processing Options dialogue to write a separate MTZ file for every block of images. These files can be used to run either *POINTLESS* or *SCALA* before the integration has finished and there is an option to run *POINTLESS* automatically after integration of each block of images.

### 6.1. Refined detector parameters

The refined detector parameters are plotted as integration proceeds. Any combination of parameters can be plotted simultaneously, although for simplicity only two different vertical scales are allowed. There is also the option to fix any of the refineable parameters at the input value, which can improve the processing of very weak diffraction data. These graphs will highlight any instability in the refinement. Typically, beam coordinates should not vary by more than 0.1 mm, detector tilt and twist should not vary by more than 0.2°, the distance should be stable to within 0.5 mm and the YSCALE value should equal 1.000 (except as noted in §[Sec sec5]5) for all images. In addition, the r.m.s. residual (the r.m.s. difference between observed and predicted spot positions) should remain approximately constant, especially for spots in the central region of the detector, unless there is noticeable change in spot shape. Typical values are 0.03–0.06 mm for synchrotron data, where the spots are usually relatively small.

If the crystal unit-cell parameters change significantly during the data collection as a result of radiation damage, the crystal-to-detector distance will decrease monotonically and the YSCALE parameter may also change. In addition, if the cell parameters are not accurate, the YSCALE and detector distance parameters will vary in a systematic way in an attempt to achieve the best fit of the predicted and observed spot positions. Inaccurate cell parameters may also result in large variations in the detector tilt and twist. This can, for example, occur when an orthorhombic solution has been chosen for a crystal that is actually monoclinic but with a β angle close to 90°. Systematic changes in the detector tilt and twist are excellent indicators of inaccurate cell parameters or other problems with the integration.

### 6.2. Refined crystal parameters

The crystal missetting angles [Φ(*x*), Φ(*y*), Φ(*z*)] are refined independently for every image (or, for fine-sliced data, for groups of images). It is not unusual to see changes of a few tenths of a degree in these angles for a complete data set. This can either reflect genuine small changes in orientation or can be a consequence of the rotation axis not being exactly orthogonal to the X-ray beam (which is an implicit assumption), which results in apparent changes in missetting angles that repeat with a periodicity of 360°. Providing that the change in orientation between successive images is less than approximately one-tenth of the crystal mosaicity, these changes in orientation will have no effect on data quality. The cell parameters are normally fixed during integration because for technical reasons there are not sufficient data available to define the values of all of the cell parameters accurately. The crystal mosaicity is refined during integration, as the mosaicity can be anisotropic and can increase as a result of radiation damage. The mosaicity refinement can be unstable, refining towards a value close to zero if there are errors in either the cell dimensions or the crystal orientation. For this reason, during the first cycle of cell refinement (see §[Sec sec5]5) the mosaicity is only refined after all the images have been integrated. If the mosaicity refinement is unstable it is advisable to fix it at the estimated value and integrate a small number of images. This will update the crystal orientation and if these images are then re-integrated the refinement is often stable and the mosaicity need no longer be fixed.

### 6.3. Intensity and other statistics

The mean value of the ratio of the intensity to its standard uncertainty [*I*/σ(*I*)] is plotted separately for all reflections and for reflections in the highest resolution bin as a function of image number for both fully and partially recorded reflections (Fig. 1 ▶, lower left graph). These plots indicate if the overall strength of diffraction is changing with time, for example owing to radiation damage or owing to crystal miscentring in the beam. The number of spatially overlapped reflections, overloaded reflections and reflections flagged as ‘bad spots’ are also plotted against image number. For a crystal with a very long axis (or axes) it may be necessary to reduce the minimum spot-separation values (calculated automatically based on spot sizes) in order to avoid large numbers of reflections being flagged as spatially overlapped at low resolution. These parameters can be changed *via* the Processing Options dialogue.

### 6.4. Central spot profile

The average spot profile for reflections in the centre of the detector is plotted for every image processed (Fig. 1 ▶), with the boundary between the peak and the background regions of the measurement box plotted as a blue outline. Problems in detector-parameter refinement often result in a deterioration in the appearance of this average spot profile. The *MOSFLM* program automatically determines the measurement box parameters, which define both the overall size of the box and the position of the peak–background boundary. Additional control over the peak–background definition, making the peak region either smaller or larger, can be achieved by varying the profile tolerance parameters in the Processing Options dialogue. For very closely spaced spots the profile tolerance values can be increased, decreasing the size of the peak, which can result in more stable refinement of the detector parameters and improved spot profiles. The minimum allowed spot separation is calculated based on the size of the peak region of the average spot profile and is updated for every block of images.

### 6.5. The standard profiles

The standard profiles for different regions of the detector are displayed for each block of images processed. The peak–background boundary is plotted as described for the central spot profile. The appearance of the standard profiles gives a very good indication of the quality of diffraction. Ideally, all the standard profiles are of a regular shape and will not show evidence of spot splitting or contamination with ice spots or other non-Bragg diffraction. When the diffraction is too weak to allow the formation of a well defined standard profile, an averaged profile is calculated by including spots from adjacent regions of the detector. The averaged profiles are indicated by a red border and the original (unaveraged) profile can be viewed by clicking on the relevant profile. Parameters con­trolling the profile averaging can be altered *via* the Processing Options dialogue.

### 6.6. Resolution-dependent statistics

The mean *I*/σ(*I*) values in different resolution ranges are plotted as a histogram for each image, with separate plots for fully recorded and partially recorded reflections. This plot can provide an estimate of the resolution limit of the data as the resolution at which the mean *I*/σ(*I*) drops to below 2.0, but the final resolution limit will depend on the multiplicity of the data and the presence of radiation damage, so this is only an approximate indicator.

### 6.7. Checking the symmetry with *POINTLESS*: QuickSymm

Once a series of images has been integrated, the QuickSymm button in the tool bar launches a run of the program *POINTLESS* (Evans, 2011 ▶) to detect Laue and space-group symmetry. The results are displayed in a web browser in graphical form, as a summary and as a full logfile using the CCP4 *Baubles* utility (Briggs & Cowtan, 2007 ▶). *POINTLESS* typically gives reliable results for the Laue group based on only a few degrees of data and so if the space group is un­known it is useful to run *POINTLESS* on a few images prior to cell refinement or integration of the full data set. It is only necessary to know the correct Laue group (not the space group) to optimize the data integration.

### 6.8. Performing preliminary scaling with *SCALA*: QuickScale

The QuickScale button will first run *POINTLESS* to determine the correct Laue group and then run *SCALA* (Evans, 2011 ▶) to scale the data in the Laue group determined by *POINTLESS*. There is no control over the input to *SCALA*, which is run with the default options. The results are also displayed in a web browser using *Baubles*. Although it may be necessary to fine-tune the *SCALA* options to obtain the best final scaling, this approach provides a very rapid and useful indicator of the data quality.

### 6.9. Processing data non-interactively

Although there are many advantages to processing data interactively, there is a significant time penalty. For straightforward data sets it is often most efficient to index and refine the cell interactively and possibly integrate a few images to ensure that there are no problems, but to carry out the integration step non-interactively as a background job. The Process button in the tool bar allows submission of a batch job *via* the drop-down button options. Selecting Batch will display a *MOSFLM* script for the processing job in a new window. This script can be run directly on the host machine or sub­mitted to a remote host on the network. Experienced users can edit the script before submission or even copy and paste it into an existing generic data-processing command script. This results in faster processing, but has the disadvantage that at present it is not possible to generate the *iMOSFLM* graphical output from batch jobs. However, many of the graphs displayed in *iMOSFLM* can also be viewed by running the *CCP*4 program *LOGGRAPH* on the summary file produced by *MOSFLM*.

### 6.10. Warning messages

A summary of the warning messages produced by *MOSFLM* is produced in a pop-up box that can be viewed by clicking on the Warnings icon at the bottom right corner of any *iMOSFLM* pane. The level of importance of the warning is indicated and further details can be obtained by double clicking on the warning or by examining the *MOSFLM* logfile.

## 7. The History pane

The History pane shows a tree structure of all operations carried out during the present session. Using the Reload option, it is possible to display the graphical output of an earlier cell refinement or integration run in the session. This pane also allows access to the *MOSFLM* logfile that contains detailed output of every step of the processing. The logfile is also written to a date-stamped file with the generic name MOSFLM_*yyyymmdd_hhmmss*.lp.

## 8. Technical description

*iMOSFLM* and *MOSFLM* run as separate processes and communication between the two is *via* TCP/IP sockets. *iMOSFLM* passes instructions to *MOSFLM* in the form of standard *MOSFLM* keywords, while the information passed back to *iMOSFLM* for storage and display is defined in extensible markup language (XML).

*iMOSFLM* is written is object-oriented Tcl/Tk and makes use of a number of extensions to the core language. Several of the widgets used are built on code provided by the Iwidgets package. The image display and the customized buttons make use of parts of the tkImg extension (for displaying the diffraction image rendered in JPEG format and the buttons either as PNG or GIFs). The results from processing and the current state of the GUI are stored in an internal tree structure using the TreeCtrl package. All the XML produced by *MOSFLM* and received through the socket by *iMOSFLM* is parsed using the tDom package.

*iMOSFLM* can be run using any version of Tcl/Tk from version 8.4, although some minor versions have particular bugs that militate against their use. For example, Tk version 8.4.13 has an error in the image-display routines that make it unusably slow. Tcl/Tk 8.5 can be used, but the extensions discussed above are not included in most distributions and thus would need to be installed separately.

## 9. Conclusions

The *iMOSFLM* interface provides an intuitive easy-to-use approach to processing diffraction data. The software is undergoing active development to improve overall performance, to allow processing of images containing multiple lattices, to provide improvements to the strategy calculations where data are collected from multiple crystals and to add new task panes to allow more control over running *POINTLESS* and *SCALA* and displaying the results from these programs. The ability to launch multiple parallel background jobs to process entire data sets very rapidly and display the results graphically is also being investigated. The software is distributed with the *CCP*4 package (Winn *et al.*, 2011 ▶) and the latest versions are available from http://www.mrc-lmb.cam.ac.uk/harry both as source code and as precompiled executables for Windows, Mac OSX and Linux platforms.

## References

[bb17] Arndt, U. W. & Wonacott, A. J. (1977). Editors. *The Rotation Method in Crystallography* Amsterdam: North-Holland.

[bb1] Briggs, P. & Cowtan, K. (2007). *CCP4 Newsl.* **47**, contribution 7.

[bb2] Campbell, J. W. (1995). *J. Appl. Cryst.* **28**, 236–242.

[bb3] Evans, P. (2006). *Acta Cryst.* D**62**, 72–82. 10.1107/S0907444905036693 16369096

[bb4] Evans, P. R. (2011). *Acta Cryst.* D**67**, 282–292. 10.1107/S090744491003982X PMC3069743 21460446

[bb5] Howard, A. J. (2000). In *Crystallographic Computing 7: Macromolecular Crystallographic Data*, edited by P. E. Bourne & K. D. Watenpaugh. Oxford University Press.

[bb6] Juers, D. H., Lovelace, J., Bellamy, H. D., Snell, E. H., Matthews, B. W. & Borgstahl, G. E. O. (2007). *Acta Cryst.* D**63**, 1139–1153. 10.1107/S0907444907045040 18007029

[bb7] Leslie, A. G. W. (2006). *Acta Cryst.* D**62**, 48–57. 10.1107/S0907444905039107 16369093

[bb8] Nave, C. (1998). *Acta Cryst.* D**54**, 848–853. 10.1107/s0907444998001875 9757100

[bb9] Otwinowski, Z. & Minor, W. (1997). *Methods Enzymol.* **276**, 307–326. 10.1016/S0076-6879(97)76066-X 27754618

[bb10] Pflugrath, J. W. (1999). *Acta Cryst.* D**55**, 1718–1725. 10.1107/s090744499900935x 10531521

[bb11] Powell, H. R. (1999). *Acta Cryst.* D**55**, 1690–1695. 10.1107/s0907444999009506 10531518

[bb12] Rossmann, M. G. (1979). *J. Appl. Cryst.* **12**, 225–238.

[bb13] Rossmann, M. G., Leslie, A. G. W., Abdel-Meguid, S. S. & Tsukihara, T. (1979). *J. Appl. Cryst.* **12**, 570–581.

[bb14] Steller, I., Bolotovsky, R. & Rossmann, M. G. (1997). *J. Appl. Cryst.* **30**, 1036–1040.

[bb15] Winkler, F. K., Schutt, C. E. & Harrison, S. C. (1979). *Acta Cryst.* A**35**, 901–911.

[bb16] Winn, M. D. *et al.* (2011). *Acta Cryst.* D**67**, 235–243.

